# A Study on the Acquirement Method of Mechanical Property Parameters in the Different Base Materials Composite Region of Clad Rebar

**DOI:** 10.3390/ma15248929

**Published:** 2022-12-14

**Authors:** Zecheng Zhuang, Zhen Li, Xuehai Qian, Jianping Tan, Lei Zeng, Yang Zhao, Yong Xiang

**Affiliations:** 1 School of Mechanical and Electrical Engineering, Central South University, Changsha 410083, China; 2 Hunan Provincial Engineering Research Centre for Laminated Metal Composites, Changsha 410083, China; 3 Technology Centre, Guangxi Liuzhou Iron and Steel Group Ltd., Liuzhou 545002, China; 4 Hunan Santai New Materials Ltd., Loudi 417000, China

**Keywords:** clad rebar, composite region, structural integrity assessment, hardness, yield strength

## Abstract

Clad rebar is one of the key structures of marine and construction services. Therefore, it is of great importance to acknowledge the mechanical property parameters of the composite region in the structural integrity evaluation of clad rebar. The different base materials of clad rebar (20MnSiV/316L steel, 35#/316L steel, 45#/316L steel, and 55#/316L steel) are researched in this study. The composite area is further refined, and simultaneously, a refinement model of the composite region of clad rebar is established. In view of the fact that a surface hardness experiment is quite easy to conduct, a proposed method consists of obtaining the mechanical property parameters of materials using the surface hardness test. The mechanical property parameters are acquired; moreover, the relationship between yield stress and surface hardness of the stainless steel clad rebar is set up. We used this method to acquire the mechanical parameters of a composite surface uneven area of clad rebar, and we established a mechanical parameters mathematics model of clad rebar, it is a significant basis for a structural integrity evaluation of cladding materials.

## 1. Introduction

The structures of steel are significant parts of infrastructures. Moreover, the failure of metal structures (such as corrosion, fatigue, etc.) is always regarded as one of the potential safety hazards in critical projects [1,2]. The clad rebar, which has corrosion resistance, high strength, and high tenacity, could prevent corrosion from the outer environment and present satisfactory property behaviors. However, the composite, which resigns between the stainless steel and different base materials, belongs to the mechanical property inhomogeneity region, which also presents the weakness zone of the mechanical property of the whole structures [3]. Nevertheless, the accurate mechanical parameters of materials are significant basic data for the integrity evaluation of actual engineering structures [4,5,6]. Thus, the study of material mechanical parameters of the composite region of clad rebar is one of the major factors of structural integrity evaluation. Furthermore, it is one of the key guarantees of safety services [7]. The consideration is taken that surface hardness could be acquired easily using a hardness test. Therefore, the established method states that the surface hardness of metal materials, used to estimate the mechanical property parameters of the composite region of clad rebar, is simple and feasible [8].

A few of studies were conducted on this issue by multiple scholars, domestic and foreign. Hamed Dabiri et al. [9] implemented Tree-Based machine learning techniques to analyze the ultimate strain of non-spliced and spliced steel reinforcements. A total of 225 experimental tests was collected and the databases were divided into training (85%) and testing (15%) of the developed models. This model can be further considered as a part of a comprehensive prediction model for estimating the stress–strain behavior of steel bars. Kyriakos I. Athukoralalage et al. [10] studied the relationship between material hardness and yield strength on different positions of the railway, and their relationship model was established and verified using existing materials of railway tracks. Daniel G. Mevec et al. [11] studied the material properties of crankshaft bearing after surface hardening. The methods of X-ray diffraction and hardness test were used to obtain the hardening depth of the crankshaft bearing after heat treatments. Andre Rudnytskyj et al. [12] investigated the relationship between the thermal viscoplastic flow stress and the hardness of 6061 and 6016 aluminum alloys during the hot rolling process. It was qualified that the hardness decreased with the fall in temperature. Meanwhile, the constraint factor diagram of the thermal viscoplastic flow stress and material hardness was given. Furthermore, the model and the constitutive equation were verified by means of the hardness test and indentation morphology. A. Brencich et al. [13] explored the material yield stress of construction rebar in service by the Richter hardness. Hence, the relationship between Richter hardness and yield strength was established and a method of moderate damage detection was proposed to acquire the parameters of the steel bar in service which was superior to the tensile test. Mudhaffar et al. [14] studied the micro-structure and mechanical properties of the stainless steel clad rebar by designing different passes and selecting different rolling process parameters. The results showed that the bimetal formed strong metallurgical bonding with better bonding performance. It was concluded that the rolling speed compared with rolling temperature, rolling direction and reduction ratio had a smaller effect on the mechanical properties of the stainless steel clad rebar. Sawicki et al. [15] placed the stainless steel rods below the non-melting electrode, and tungsten inert gas shielded welding was used to generate a high temperature to melt the stainless steel rods and cladding spray onto the carbon steel surface. However, some problems remain in this process, such as uneven distribution of stainless steel cladding and difficulty in biting into the rolling mill. Visar Farhangi et al. [16] analyzed the structural responses of glass-fiber-reinforced polymer tubes, investigated the lateral strength of the GFRP composite pile and pre-stressed piles under both axial compression and bending moment loads; the required bending and corrosion resistance capacities of piles in different ranges of eccentricities can be reached using the combination of tube wall thickness and GFRP fiber percentages.

Domestically, Ying-Ying Feng et al. [17] simulated a unidirectional compression test of stainless steel clad rebar by MMS-200 thermodynamic simulator, under different compression temperatures (950 °C, 1050 °C) and compression rate (50%, 70%), respectively. The research showed that a small amount of manganese silicon oxide and manganese sulfur inclusions were dispersed on the composite surface when the rolling temperature was 1050 °C and the compression rate was 70%. moreover, the tensile strength and elongation of the clad steel bar reach 690 MPa and 26%, respectively, which far exceeding the specified value. Liu et al. [18] used the flux deposition process to prepare composite billet. The process effectively solved the interface oxidation problem of billet during heating and rolling, however, the production cost was high. Hao F. et al. [19] studied the relationship between the micro-hardness and yield strength of oxygen content on the metal surface of Ti6Al4V. It was found that when the material’s depth increased, the oxygen content, hardness, and yield strength all decreased and the linear relationship between them remained. Furthermore, a linear relationship was discovered between hardness and yield strength through the experiment of the SODH metal layer. Si G. et al. [20] researched the relationship among the residual stress, hardness, and strength of aluminum alloy 2024-T4 laser welded joints, and the Mises stress was similar to the yield stress in the joints regio. Moreover, the hardness decreased on account of the residual stress influence on the aluminum alloy 2024-T4 cladding zone. Finally, the sequence prediction model was set up. Hong Zhou et al. [21] studied the EH47 steel with high tensile strength; the steel plates were welded by means of a multi-pass submerged arc welding method, and a micro-hardness test was conducted. The thermal elastoplastic finite element model was calculated using methods of hardening algorithm and microscopic morphology evolution. Furthermore, the hardness of FEM and a physical test were well-fitted. Hence, the influence mechanism of thermal cycling was clarified. Yu et al. [22] studied the distribution law of stress and strain of different process parameters on the reinforcement of cladding by using the Mark software; meanwhile, the relationship was established between the thickness of stainless steel tube and the minimum value of cladding. The results showed that the minimum value of cladding will be greater than 1 mm when the thickness of a stainless steel tube is 3~5 mm. In this paper, we established a method to obtain the material mechanical parameters in a nondestructive way, we always use standard samples to take tensile experiment, it needs to take samples from engineering structures, in this way the structure integrity could be destroyed. Thus, we want to establish a method to bridge the hardness and material parameters, so that we could obtain the material parameters by hardness test on the surface of engineering structures. Besides the clad rebar, there are some cladding material structures, such as clad section steel, clad cable, clad wire rod and clad sucker rod etc., so establishing a relationship between hardness and yield stress could play a significant role in cladding structure integrity evaluation and in service life prediction.

## 2. Theoretical Basis

Austenitic stainless steel and carbon steel are widely used in construction structures, due to their good mechanical behaviors of strength toughness and corrosion resistance. They generally belong to power-law hardening materials. Moreover, their mechanical relationship is expressed by the Ramberg–Osgood (R–O) stress–strain relation. Therefore, the different composite regions of clad rebar could be regarded as a power-law hardening material, which conforms to the R-O relation when the FEM was processed [23].

(1) $$\frac{\varepsilon }{\varepsilon_{0}}=\frac{\sigma }{\sigma_{0}}+\alpha {\left(\frac{\sigma }{\sigma_{0}}\right)}^{n}$$

where *α* is the migration coefficient, *n* the hardening index and *σ* and *ε* the true stress and strain, respectively. *σ*_0_ and *ε*_0_ are the yield stress and yield strain, respectively.

Regarding the hardening index, the relations are as follows [24]:

(2) $$n=\frac{1}{\kappa \ln\left(1390/\sigma_{0}\right)}$$

where κ = 0.163. Since the hardness and yield strength of different composite areas of clad steel are different, the hardening index n calculated by Equation (2) is different as well.

The Vickers hardness value calculation formula is as follows [25]:

(3) HV=0.102FS=0.1022Fsin(θ2)d2=0.1891Fd2

where *F* is the indentation load, *S* the indentation area, *θ* the relative angle between two indentation head surface, and *d* the average indentation diagonal length.

*d* is related to the indentation depth h:

*d* = h·tan68° (4)

The relation of material Vickers hardness HV and indentation depth:

(5) $$\mathrm{HV}=\frac{0.3025}{h}$$

## 3. Materials and Experimental Method

### 3.1. Clad Rebars Rolling Process and Chemical Composition

**Figure 1:**
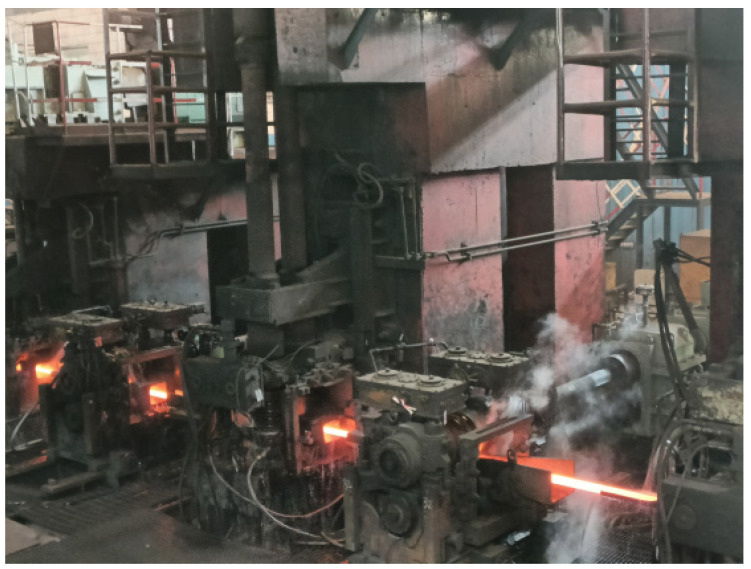
Rough rolling process in factory.

Clad rebars were researched in this study and were hot-rolled through 14 passes according to the hot-rolling process in a steel factory in Guangxi, China. The rough rolling stages are shown in Figure 1.

**Figure 2:**
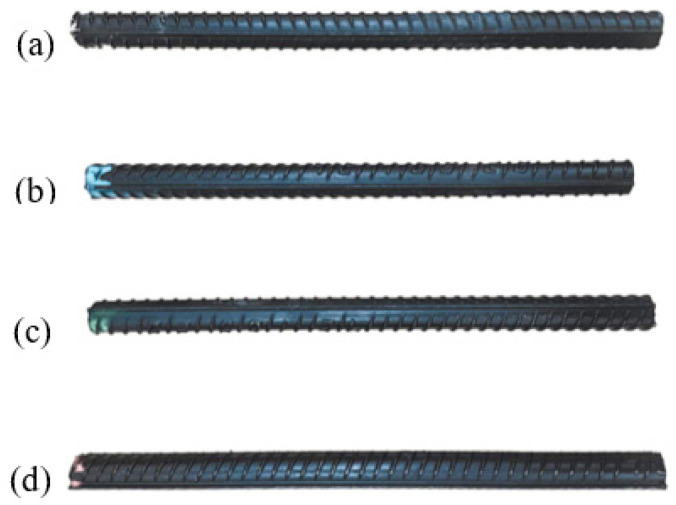
Clad rebar of different base metal: (**a**) 316L SS–20MnSiV clad rebar; (**b**) 316L SS–35# CS clad rebar; (**c**) 316L SS–45# CS clad rebar; (**d**) 316L SS–55# CS clad rebar.

The cladding material of clad rebar is the 316L austenitic stainless steel. The base metals are 20MnSiV, 35# steel, 45# steel, and 55#steel different carbon steels, as shown in Figure 2. Thereinto, Figure 2a represents the 316L SS-20MnSiV clad rebar, Figure 2b the 316L SS-35# CS clad rebar, Figure 2c the 316L SS-45# CS clad rebar, and Figure 2d the 316L SS-55# CS clad rebar. The chemical composition of different carbon steel base metals and the 316L SS cladding material are shown in Table 1. A large difference remains between carbon steel and stainless steel, especially considering the elements of Fe, Ni, and C.

### 3.2. Experimental Methods

**Figure 3:**
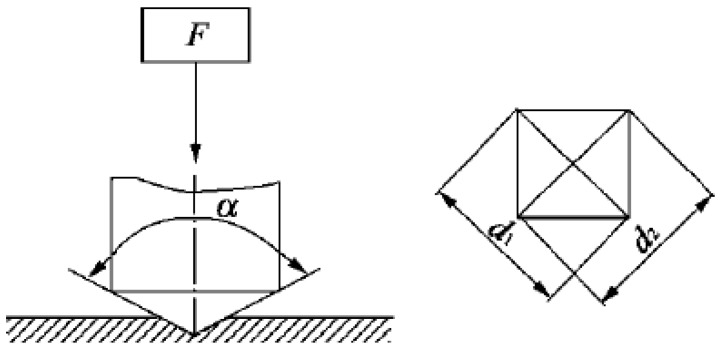
Principle of indentation test.

The samples were polished, and the micro-indentation test was carried out using a Shimadsu HMV-2T micro-hardness tester to obtain the indentation morphology and hardness gradient curve of each region of clad rebar. The micro-structure morphology of the composite region of clad rebar was observed via a LEICA DM4M metallographic microscope. The indenter of the micro-hardness test, whose angle is 136°, is made by a regular tetrahedral diamond, as shown in Figure 3. The experiment loads were applied on 0.49 N, 0.98 N, 1.96 N, 2.94 N, 4.9 N, and 9.8 N. Meanwhile, the test force was loaded and unloaded automatically, the loading time was generally 15 s. The hardness values were calculated by the indentation diagonal length after the test.

**Figure 4:**
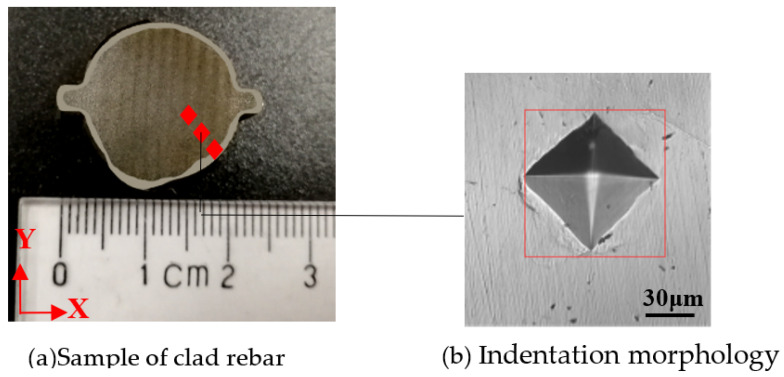
Clad rebar sample and indentation morphology.

The metallographic sample was ground using an automatic grinding machine, the Shimadsu HMV-2T micro-hardness tester was used to take an indentation test. Samples were made from different base material and stainless steel of 316L SS-20MnSiV, 316L SS-35# CS, 316L SS-45# CS, 316L SS-55# CS; the sample was as shown in Figure 4a. First of all, the indentation test was conducted on the carbon steel base material and stainless steel cladding material at room temperature, and the distance between the two indentations was 10 µm, Three sets of tests were performed at each test range and indentation diagonal dimensions and hardness values were recorded. Simultaneously, since the metallurgical bonding surface of the clad rebar is small, the test range was selected as 0.245 N, the distance between the two indentations was 10 µm, indentation test was carried out along 316 SS to carbon steel, and the hardness ingredient curves of each region of clad rebars were obtained, the hardness test site was as shown in Figure 4a. Furthermore, each experiment force was tested three times, the red points represented indentation test track and the indentation morphology was measured by a rectangular frame from the outside. The indentation morphology after the test was as shown in Figure 4b.

## 4. Finite Element Analysis

### 4.1. Dimensions Selection

The ABAQUS, large nonlinear finite element software, was used for numerical simulation calculation and analysis in this paper, however, there is no fixed dedicated dimensional system in ABAQUS, so we chose SI/mm–N–Tone–MPa dimensional system according to the factors of actual calculation requirements and model geometric parameters. The common dimension systems were as shown in Table 2.

### 4.2. Geometry and Material Model Establishment

**Figure 5:**
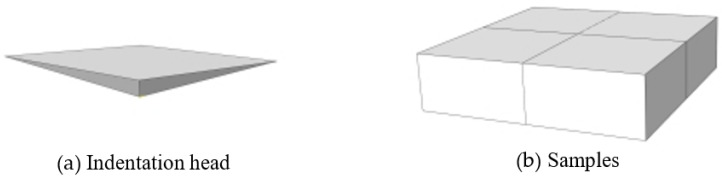
Geometry model of FEM.

The FEM model was established to compare with a physical test, therefore, the subject of the hardness test was a plat; the indentation head is a rectangular pyramid of 2 mm × 2 mm, as shown in Figure 5a. The FEM model was simplified to a cuboid of 40 mm × 40 mm × 3 mm as shown in Figure 5b. The stainless steel and carbon steel model conforms to the R–O relationship by virtue of which they belong to the power-law hardening materials.

### 4.3. Mesh Model and Assembly

**Figure 6:**
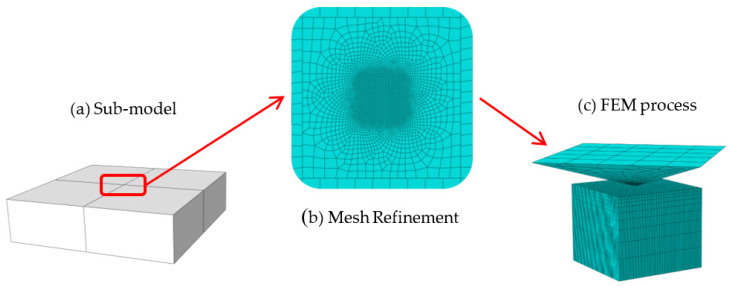
FEM model establishment.

The sample of FEM was as shown in Figure 6a, the mesh refinement was as shown in Figure 6b, and the assembly was as shown in Figure 6c. The sub-model, a cuboid of 1 mm × 1 mm × 0.3 mm, was used on the key part of FEM model and the critical part to mesh refinement after the whole model was calculated; C3D8R (Continuum 3 Dimensional 8 node, Reduced integration) was adopted as the mesh type. Meanwhile, the sub-model was cut out for analysis from the key part of the contact between indentation head and sample. The indentation head was treated as a rigid body. The total numbers of model units were 80,190, the total numbers of sub-model units were 65,530, finally, the sample and indentation head were assembled, the FEM model establishment was shown in Figure 6.

### 4.4. Loads and Boundary Conditions

The indentation load was 9.8 N in the indentation experiment, the uniform pressure was set up 2.45 MPa on the indentation head, the loading time was 15 s, the finite element model was set to calculate yield strength of materials at a certain interval value. The indentation size corresponding to the strength was obtained and compared with the physical experiment results. The accuracy of material parameters were verified on the comparison between indentation test and numerical simulation under different loads of 0.49 N, 0.98 N, 1.96 N, 2.94 N, 4.9 N and 9.8 N. Boundary conditions were set to be completely constrained on the lower plate surface, and the indentation head was allowed in Y direction movement degrees of freedom. The interaction was divided into two periods: before indentation test and indentation test process; in the period of before indentation test, the bottom surface was selected as master surface of contact, the top surface of sample was selected as slave surface of contact. The contact property was tangential behavior, the friction formulation was penalty, the directionality was isotropic and the friction coefficient was 0.2.

## 5. Results and Discussions

### 5.1. Experimental Results

**Figure 7:**
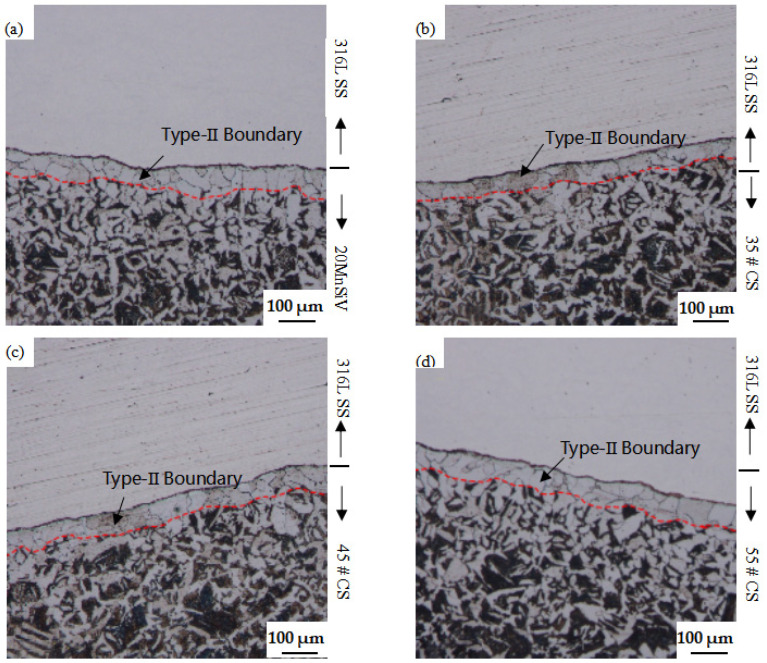
Microscopic morphology of 20MnSiV, 35#, 45#, 55# different carbon steel: (**a**) 20MnSiV–316L SS clad rebar; (**b**) 35# CS–316L SS clad rebar; (**c**) 45# CS–316L SS clad rebar; (**d**) 55# CS–316L SS clad rebar.

The grain boundary structure of the poor carbon zone and the fusion boundary resulted from the process of hot rolling. The material chemical composition and the micro-hardness were both changed, which made it difficult to determine the material properties in each sub-region of the composite region [26]. The different base metals of carbon steel and austenitic stainless steel of cladding material were as shown in Figure 7; the metallographic micro-structures of clad rebar of the 316L SS cladding material, as well as 20MnSiV, 35#, 45#, and 55# different base metals of carbon steel were as shown in Figure 7a–d. It is found that the composite surface of the clad rebar has a satisfactory bonding performance, and there are no cavities, cracks, or oxides, which is attributed to the cleaning treatment of the billet surface before assembly.

The micro-structure of the carbon steel side shows a narrow region with a width of approximately 120 μm near the fusion boundary (FB), and obvious dendrite structures are remaining, containing the type I grain boundary perpendicular to the FB line, and type II grain boundary parallel to the FB line, in addition to high hardness and high residual strain, as shown in Figure 7. The carbon element is diffused from the bottom layer into the cladding material, and the carbon steel side is decarbonized during the formation of the clad rebar. With the increase of the carbon content in the base metal, the width of the decarbonization zone expands to 50 µm~60 µm. Moreover, this region becomes the dilution zone. This unique grain boundary in the fusion boundary of carbon steel results from the obvious change of micro-structure and composition, stressing the fact that the corrosion crack (SCC) is easily expanded along this path. The large angle grain boundary of type II has a higher SCC sensitivity than type I [27,28].

**Figure 8:**
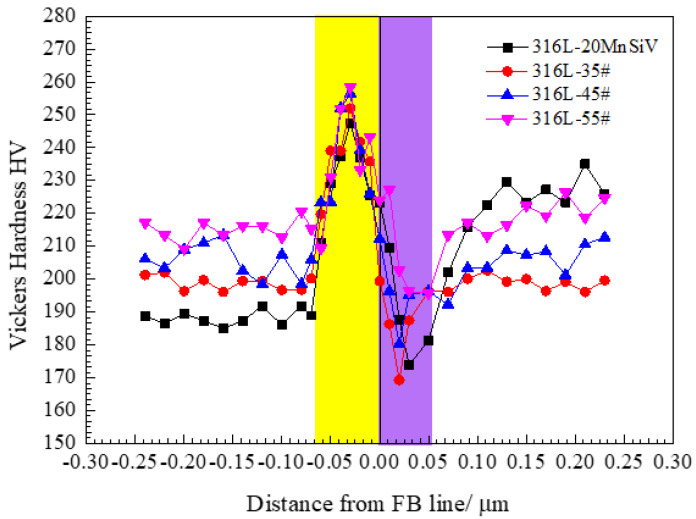
Microhardness gradient curve of 20MnSiV, 35#, 45#, 55# CS, and 316L SS clad rebar.

The micro-hardness test was carried out to obtain the hardness gradient curve along the 316LSS to the carbon steel side to investigate the mechanical properties of the composite region between different kinds of carbon steel and stainless steel. The gradient curve is shown in Figure 8.

The metallographic micro-structure of different base metals and stainless steel, and the hardness gradient curve of clad rebar are shown in Figure 7 and Figure 8. The traditional ‘sandwich model’ of clad rebar is further refined into Cladding Material (CM), High Hardness Zone (HHZ), Dilution Zone and (DZ) Base Metal (BM). The HHZ zone is shown in the yellow part of Figure 7 (the left part of the FB line) while the DZ zone is in purple (the right part of the FB line). The summit value of hardness occurs in the vicinity of the fusion boundary of the composite area, within a narrow HHZ region. Meanwhile, the micro-hardness here is the maximum and the hardness appears in a gradient change, obviously. Furthermore, the gradient changes of mechanical properties exist near the FB line and the DZ area between the base metal and the cladding material; the structures of the composite transition zone are relatively complex, which could significantly affect the SCC sensitivity of the composite region. This was confirmed by the study of Yingying Feng et al. and Bulent Kurt et al. [9,29]. A bright-field TEM was used to observe that the striped martensite structure was produced on the side of the austenitic stainless steel, according to Li Zhen et al. [30]. The martensite oversaturated carbon and the lattice distortion appeared. Simultaneously, the dispersed carbide was separated out, and the strengthening solid solution was generated. Moreover, a large number of crystal defects and micro-structure refinement were caused during the transformation of martensite, which could strengthen the martensite and present a satisfactory hardness behavior.

The mechanical properties of different base metals of the clad rebars are shown in detail in Table 3. Zhen Li et al. found that the material properties of clad rebar are better than the single cladding material or the single base material [31].

### 5.2. Simulation Results

**Figure 9:**
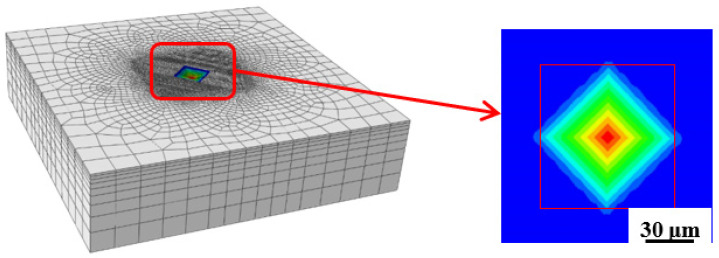
The indentation results of FEM.

The FEM results, under different indentation loads where the yield stress of the material was 390 MPa are shown in Figure 9.

The diagonal length of indentation under different yield stresses is shown in Table 4. It is found from Table 4 and Table 5 that when the indentation load is 9.8 N and the yield stressσ0 is 390 MPa, the hardness of FEM is the most consistent with the micro-hardness test. These results are also found in the comparison between Figure 4 and Figure 9.

### 5.3. Comparison of Experiment and Numerical Simulation Results

**Figure 10:**
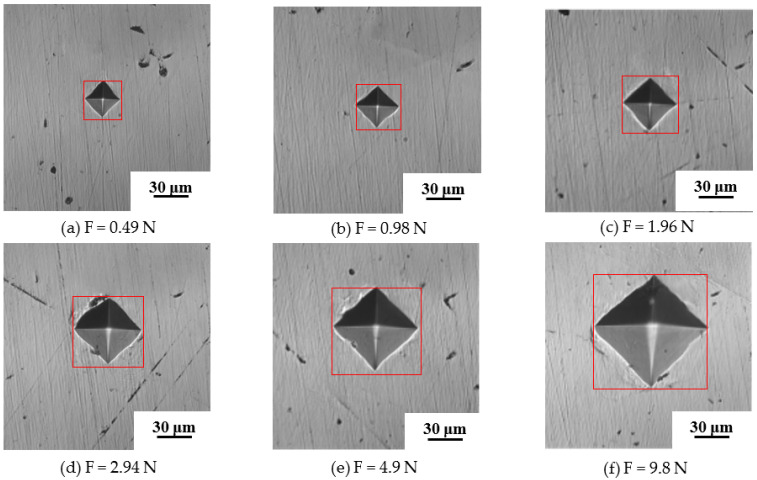
Indentation results under different indentation loads of 20MnSiV.

The micro-hardness test was conducted under different loads of the indentation from 0.49 N to 9.8 N, and the results are shown in Figure 10.

The experimental data are shown in detail in Table 5. It is found that the diagonal indentation length increases with the increase of the indentation pressure; however, the hardness is basically constant. Thus, it is determined that the hardness value of the material has nothing to do with the indentation’s load within a certain range, which also fits the experimental standards [25].

**Figure 11:**
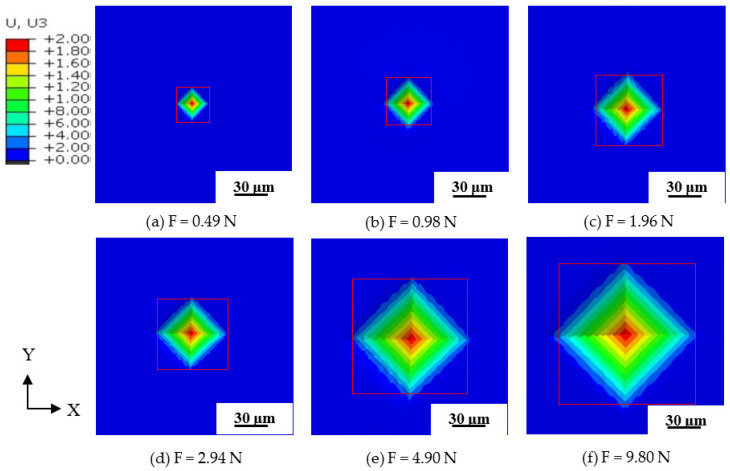
FEM results under different indentation pressure of 20MnSiV.

The FEM results under the indentation’s pressure of 390 MPa are shown in Figure 11, and it is clear that with the increase of the indentation’s pressure, the diagonal indentation length augments. The data of FEM results are shown in detail in Table 6.

The relationship between the surface hardness and the diagonal length of indentation is obtained via the micro-hardness test. Meanwhile, the relationship between the yield stress and diagonal length of indentation is acquired using the FEM. Thus, the diagonal length of indentation could be helpful to establish the relationship between the surface hardness and yield stress of experimental materials.

The diagonal indentation length under different indentation pressures of FEM is shown in Figure 11, as the material yield stress is 390 MPa. The indentation results show that the diagonal length of the indentation is directly proportional to the indentation pressure, and the results of the hardness experiments and FEM fit well. It is indicated that the combination of the hardness test and FEM to obtain the yield stress of the material is feasible. Furthermore, this was verified by Kyriakos I. et al. and A. Brencich et al. [10,13].

### 5.4. Acquisition of Mechanical Property Parameters of Materials in Composite Region

The traditional model was refined and the refinement model of clad rebar of Cladding Material (CM), High Hardness Zone (HHZ), Dilution Zone(DZ), and Base Metal (BM) was established according to the micro-morphology and micro-indentation of clad rebar. The HHZ was approximately 60~70 μm and the DZ was approximately 50~60 μm along the fusion boundary direction. The yield stress of the non-uniform region of the composite area is obtained, as shown in Table 7.

It can be found that the hardness and strength of 35# steel is minimal. However, the hardness and strength of 55# steel are clear in the following ranking of the hardness value: 35# steel < 45# steel < 55# steel < 20MnSiV. For single material, the hardness and strength values of the DZ material are the lowest, the hardness and strength values of the HHZ are the highest, and the gradient curve of hardness appears on the clad rebar; hence, the necessity of establishing a refinement model of the composite’s non-uniform region is further verified. Furthermore, with the increase of the carbon content, the hardness and strength of 316L SS of different types of clad rebar increase as well, which verifies that the carbon element migrated from the carbon steel side to the austenitic stainless steel side. Simultaneously, the poor carbon zone appears on the carbon steel side as shown in Figure 7, the carbon content of austenitic stainless steel increases, as well as the hardness and strength.

**Figure 12:**
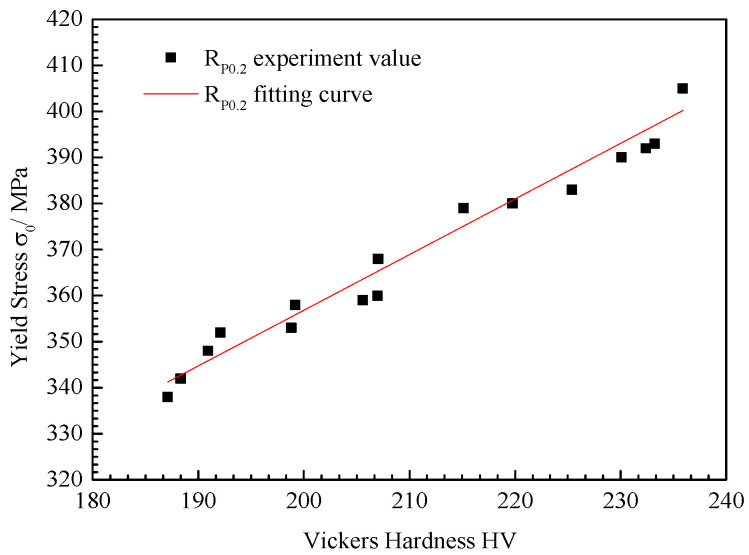
Fitting curve of microhardness and yield stress of clad rebar.

The fitting curves of microhardness and strength in each region of the non-uniform mechanical properties were obtained using the FEM numerical simulation and indentation test, as shown in Figure 12. Since no obvious yield platform appeared in the tensile curve of austenitic stainless steel, the specified non-proportional tensile strength R_P0.2_ was used to represent the yield strength σ_Y_. The yield strength and microhardness in each area of the composite region of clad rebar were obtained via linear fitting between the finite element numerical model and the indentation test: R_p0.2_ = A *Hv + B, A = 1.21, B = 115.19, and the correlation coefficient R^2^ = 0.98.

## 6. Conclusions

(1) A refinement model of the composite region of clad rebar was established, and the traditional ‘sandwich model’ of cladding material-composite surface-base metal was further refined: Cladding Material (CM), High Hardness Zone (HHZ), Dilution Zone(DZ), and Base Metal(BM). Moreover, the HHZ region ranged from 60~70 μm and the DZ region ranged from 50~60 μm;
(2) The proposed method aims to obtain mechanical properties of the composite region of clad rebar by using the surface hardness. The hardness and yield stress of 20MnSiV#DZ is 190.92 HV, 315 MPa; the hardness and yield stress of 20MnSiV#HHZ is 230.09 HV, 400 MPa; the hardness and yield stress of 35 CS#DZ is 187.10 HV, 295 MPa; the hardness and yield stress of 35 CS#HHZ is 232.40 HV, 405 MPa; the hardness and yield stress of 45 CS#DZ is 192.09 HV, 320 MPa; the hardness and yield stress of 45 CS#HHZ is 233.23 HV, 410 MPa; the hardness and yield stress of 55 CS#DZ is 207.03 HV, 360 MPa; and the hardness and yield stress of 55 CS#HHZ is 235.88 HV, 415 MPa. It could be used to roughly obtain the basic mechanical properties of the composite region of clad rebar;
(3) The yield stress values of different clad rebar were acquired, and according to the material parameters of each uneven region of a different carbon steel base metal of clad rebar, the mechanical parameters model of the clad rebar was: R_p0.2_ = A *Hv + B, A = 1.21, B = 115.19, and the correlation coefficient was R^2^ = 0.98. Furthermore, this linear relation could provide a certain scientific basis for the structural integrity evaluation of clad rebar.

## Figures and Tables

**Table 1:** Chemical composition of base metal and cladding metal (ωt%).

Material	C	Si	Mn	P	S	Cr	Ni	Mo	Fe
20MnSiV	0.22	0.35	1.37	0.008	0.005	0.20	0.04	/	Bal.
35# CS	0.35	0.34	0.75	0.009	0.004	0.21	0.05	/	Bal.
45# CS	0.46	0.33	0.65	0.013	0.002	0.18	0.05	/	Bal.
55# CS	0.53	0.28	0.55	0.011	0.006	0.19	0.04	/	Bal.
316L SS	0.021	0.65	0.81	0.018	0.008	17.9	13.5	2.1	Bal.

**Table 2:** Common dimension systems.

Dimensions	SI	SI/mm	US Unit/ft	US Unit/Inch
Length/l	m	mm	ft	in
Force/F	N	N	lbf	lbf
Mass/m	kg	tonne(10^3^ kg)	slug	lbf s^2^/in
Time/t	s	s	s	s
Stress/σ	Pa(N/m^2^)	MPa(N/mm^2^)	lbf/ft2	Psi(lbf/in2)
Energy/Q	J	mJ(10^−3^J)	ft lbf	in lbf
Density/ρ	kg/m^3^	tonne/mm^3^	slug/ft3	lbf s^2^/in

**Table 3:** Results of tensile experiments on rebars.

Rebar Type	Yield Stress (MPa)	Tensile Strength (MPa)	Elongation (%)
20MnSiV/316L	455	628	26.3
35#/316L	335	575	23.6
45#/316L	363	638	16.2
55#/316L	380	650	14.2

**Table 4:** Indentation diagonal length of numerical simulation under different yield stress.

Yield Stress σ_0_/MPa	360	370	380	390	400	410	420
Indentation Diagonal d/µm	88.66	90.89	93.21	95.00	97.26	98.36	100.40

**Table 5:** Results of Vickers hardness test under different indentation loads.

F/N	d_1_/µm			d_2_/µm			h/µm			$\bar{d}$/µm	HV
0.49	21.11	20.11	21.85	20.85	20.88	21.08	4.24	4.14	4.34	20.98	214.59
0.98	30.64	29.41	30.12	29.61	30.61	29.84	6.09	6.06	6.06	30.04	213.39
1.96	40.42	40.39	40.39	41.19	40.19	40.65	8.24	8.14	8.19	40.54	212.71
2.94	52.01	50.72	52.69	52.95	53.29	52.18	10.60	10.51	10.59	52.31	205.68
4.90	67.93	67.14	66.15	67.42	67.15	66.38	13.67	13.56	13.39	67.03	202.37
9.80	97.32	94.49	94.23	95.00	95.00	95.77	19.43	19.14	19.19	95.30	200.54

**Table 6:** Results of numerical simulation under different indentation loads.

F/N	0.49	0.98	1.96	2.94	4.90	9.80
d/µm	18.64	27.62	38.55	51.33	66.57	94.89
h/µm	7.52	11.14	15.54	20.70	26.84	38.26

**Table 7:** Mechanical parameters of non-uniform region of clad rebar.

Materials	20MnSiV	20MnSiV#DZ	20MnSiV#HHZ	316L
Hardness HV	225.38	190.92	230.09	188.33
Yield Stress σ_0_/MPa	390	315	400	300
Materials	35#	35#DZ	35#HHZ	316L
Hardness HV	199.17	187.10	232.40	198.80
Yield Stress σ_0_/MPa	320	295	405	330
Materials	45#	45#DZ	45#HHZ	316L
Hardness HV	206.98	192.09	233.23	205.58
Yield Stress σ_0_/MPa	355	320	410	350
Materials	55#	55#DZ	55#HHZ	316L
Hardness HV	219.75	207.03	235.88	215.11
Yield Stress σ_0_/MPa	375	360	415	360
